# Pharmacokinetics of furosemide in thoroughbred horses subjected to supramaximal treadmill exercise with and without controlled access to water

**DOI:** 10.1186/s12917-019-2017-3

**Published:** 2019-08-02

**Authors:** N. F. Villarino, C. M. Lopez, R. A. Sams, W. M. Bayly

**Affiliations:** 1 Program in Individualized Medicine, Department of Veterinary Clinical Sciences, Washington State University, Pullman, WA 99164-6610 USA; 2 Department of Veterinary Clinical Sciences, Washington State University, PO Box 646610, Pullman, WA 99164-6610 USA; 3 LGC Science Inc., Lexington, KY 40509 USA

**Keywords:** Diuretics, Controlled water intake, Exercise-induced pulmonary hemorrhage, Furosemide, Pharmacokinetics, Horses

## Abstract

**Background:**

The primary objective of this study was to assess the disposition of furosemide in Thoroughbred horses treated intravenously with 1 mg/kg of furosemide 4 and 24 h before supramaximal treadmill exercise without and with controlled access to water, respectively. Another objective was to determine whether furosemide was detectable in the plasma of horses after exposure to supramaximal treadmill exercise. Thoroughbred horses (*n* = 4–6) were administered single intravenous doses of 1 mg/kg of furosemide at 4 and 24 h before supramaximal exercise on a high-speed treadmill, with controlled and free access to water, respectively. Plasma furosemide concentrations were determined using liquid chromatography.

**Results:**

Furosemide was detected in all the horses, regardless of whether they were treated 24 h or 4 h before excersice. In both treatment sequence groups of 2 horses, the concentration time profiles of furosemide during the first 4 h after its administration were relatively similar. The average maximum observed concentrations, AUC_0–1.5h,_ and AUC_0–3h_, of both groups of horses were not different (*p* > 0.05). There were no significant differences in systemic clearance based on the geometric mean (95% confidence interval) (409 (347–482) mL/h/kg) for 4 h and 320 (177–580) mL/h/kg) for 24 h) between horses that were exercised 4- and 24-h post-furosemide administration. The plasma concentration of furosemide in all the horses fell below the limit of quantification (25 ng/mL) within 12 h after drug administration. In the group treated 24 h before exercise, none of the horses had detectable furosemide at the time of supramaximal treadmill exercise. In the group treated 4 h before exercise, furosemide was detected 1 h before and 2 h after supramaximal treadmill exercise in 4/4 and 3/4 horses, respectively. The mean AUC_3-last h_ of both groups of horses were not different (*p* > 0.05).

**Conclusions:**

Water restriction did not exert any apparent effect on the disposition of furosemide. It remains to be determined, however, whether the attained plasma concentration of furosemide in combination with other controlled water access protocols have any direct or indirect pharmacological effect that may affect the athletic performance of the horse.

## Background

Furosemide is a high-ceiling loop diuretic. In horses, its plasma concentration decreases rapidly following intravenous administration and this is reflected by its high clearance (8.9–10.7 mL/min/kg) in exercised horses [1] and short terminal half-life (2–4 h) [2]. In horses, 50–60% of a furosemide dose is cleared renally, whereas the rest is conjugated to glucuronic acid in the liver or in the kidneys [3] and excreted in the urine. In healthy horses with free access to drinking water, at least 90% of the intravenous dose of furosemide is eliminated within 4 h following drug administration. The diuretic effect of furosemide is correlated with its plasma concentration. In fact, this drug produces a marked increase in the rate of urine formation between 15 and 30 min following its intramuscular or intravenous administration. However, no diuretic effect can be detected, 4 h after its administration [4].

Furosemide is administered approximately 4 h before racing in accordance with the rules of racing in the USA and numerous other countries in the Americas. Its use came to prominence in the late 1960s and early 1970s for its putative role in the prophylaxis of exercise-induced pulmonary hemorrhage (EIPH) [5–8]. EIPH is bleeding in the lungs that occurs in the majority of Thoroughbred and Standardbred racehorses subjected to strenuous exercise [9]. It has been suggested that furosemide attenuates the severity of EIPH by reducing pulmonary vascular pressures during strenuous exercise, although a mechanism by which the drug does this has yet to be identified.

At racetracks where the rules of racing allow its use, furosemide is administered intravenously to horses at a dose up to 0.5 or 1.0 mg/kg body weight, depending on the racing authority. However, national and international pressure has been growing to prohibit race day medication in order to avoid any potential beneficial effect of furosemide which might directly or indirectly alter the athletic performance of the horse, and the resultant negative public image of the sport that stems from this possibility [9]. Unfortunately, furosemide is the only pharmacologic treatment for EIPH that has been scientifically demonstrated to mitigate EIPH. Therefore, if race day administration of furosemide is prohibited, veterinarians may start administering furosemide to horses on the day before racing. Consequently, the pharmacokinetics of these dosage regimens when given this far before racing warrant investigation, particularly when the possible effects of strenuous exercise and pre-race management practices such as controlling access to water are considered.

Administration of furosemide is not the only management strategy used to prevent or reduce the severity of EIPH in horses. Controlled intake of water before racing is also sometimes practiced in the belief that it helps lower blood pressure, and therefore prevents or decreases the severity of EIPH [10]. Although several studies have been carried out to assess plasma disposition of furosemide, the kinetics of this drug in horses subjected to a controlled intake of water for 24 h remains to be determined. In fact, in strenuously exercising horses, managed water intake and a marked contraction of the circulating plasma volume subsequent to furosemide administration [11] could alter the fate of furosemide. A decrease in the rate of elimination of the drug could result in an increase in its plasma concentrations and this in turn could prolong the overall pharmacological effect of the agent. It might also increase the likelihood of the drug being detected post-racing in jurisdictions in which the presence of any furosemide in a test sample collected from the horse is a punishable offence. Using an elegant experimental design, Knych and colleagues reported no significant differences in the volumes of distribution at steady-state or systemic clearances between horses that were exercised at 4 and 24 h following the iv administration of 250 mg/kg of furosemide with no access to water for 4 h before treadmill exercise [12]. It is unknown whether controlling or withholding water access for longer periods than 4 h would have any effect on the disposition of furosemide.

Prolonged submaximal exercise was not found to have any apparent effect on the disposition of furosemide during the first 2 h following drug administration [1]. However a brief period of intense exercise may reduce the systemic clearance of furosemide as a consequence of decreased renal [13, 14] or hepatic [3] blood flow during the short period of exercise, thus altering the disposition of drugs cleared by the kidneys and the liver [15, 16].

Considering the gaps in our current knowledge concerning the disposition of furosemide in racehorses, one objective of this study was to assess the disposition of this diuretic in the plasma of Thoroughbred horses treated intravenously with 1 mg/kg furosemide 4 and 24 h before supramaximal treadmill exercise, without and with controlled access to water, respectively (Objective 1). Another objective (Objective 2) was to determine whether furosemide was detectable in the plasma of horses after they were subjected to supramaximal treadmill exercise under these conditions.

## Results

The administration of furosemide combined with controlled access to water and high intensity exercise protocols to fatigue had no negative impact on any of the horse health parameters investigated in this study.

### Objective 1: disposition of furosemide in the plasma of horses treated 4 and 24 h prior to supramaximal treadmill exercise

**Fig. 1:**
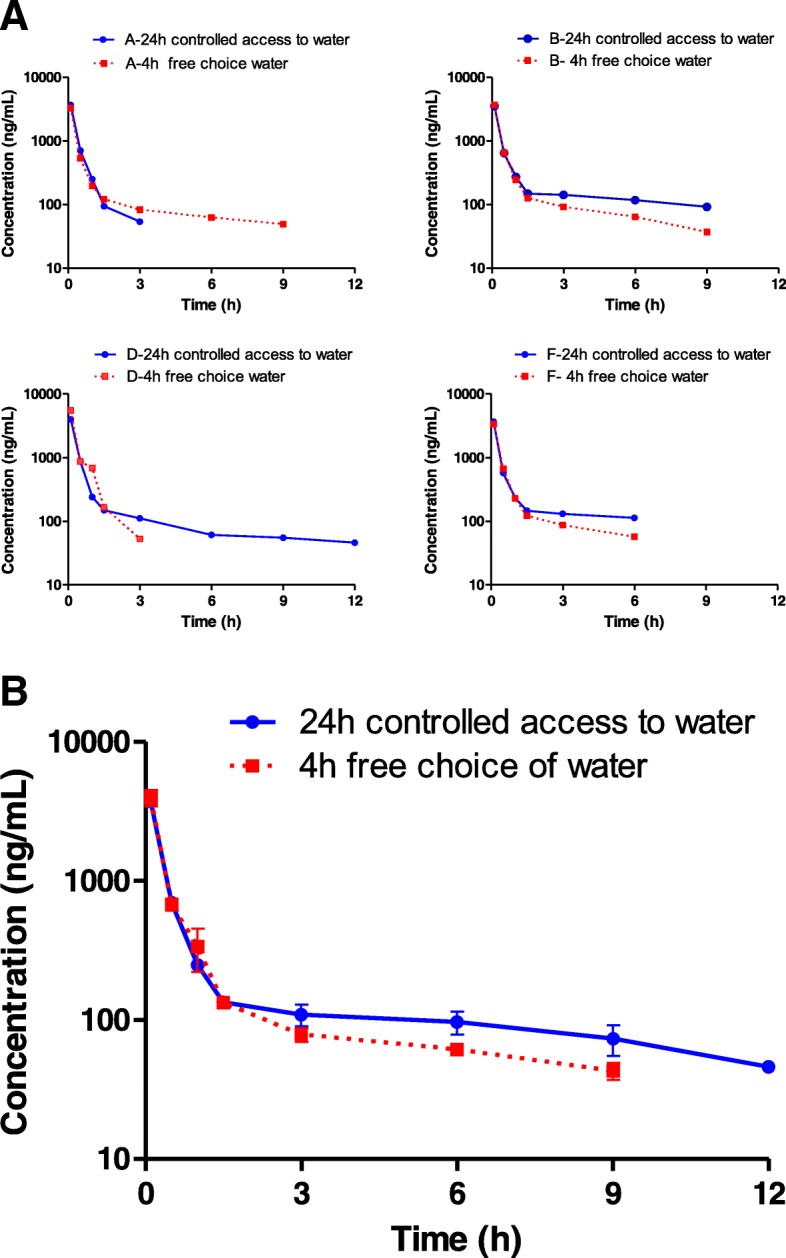
Plasma concentration of furosemide vs. time in horses treated with furosemide at 1 mg/kg of body weight intravenously 24 and 4 h pre-exercise with controlled intake of water (6 mL/kg every 4 h) with the last drink being taken 8 h before exercise (circle) and free choice of water until 4 h before exercise (square), respectively. **a** Each plot corresponds to an experimetnal horse (from A to D) and **b** mean (± SEM) plasma concentrations (*n* = 4)

The individual treadmill speeds at which the horses galloped ranged from 12.3 to 14.2 m/s, with the duration of exercise at these speeds ranging from 85 to 120 s. Furosemide was detected in all the horses, regardless of whether they were treated 24 h or 4 h before exercising (Table 1, Fig. 1). In both groups of horses, the plasma concentration of furosemide decreased at two apparent rates. Within the first 2 h following the administration of the drug, plasma concentrations decreased rapidly, whereas at a later time point the plasma concentration of furosemide decreased at a slower rate (Fig. 1).

**Table 1:** Plasma concentration (ng/mL) of furosemide in plasma from horses treated intravenously with furosemide at 1 mg/kg (*n* = 4)

Time (h)	Horses treated with furosemide 24 h before supramaximal treadmill exercise with controlled access to water				Time (h)	Horses treated with furosemide 4 h before supramaximal treadmill exercise with controlled access to water			
	A	F	B	D		A	F	B	D
0	0	0	0	0	0	0	0	0	0
0.1	3678	3666	3525	4021	0.1	3274	3369	3697	5532
0.5	707	574	645	871	0.5	534	669	651	876
1	250	232	272	241	1	197	228	243	685
1.5	94	147	148	149	1.5	121	122	126	166
3	54	131	142	111	3	83	87	92	53
6	ND	113	117	61	6	63	57	64	ND
9	ND	ND	92	55	9	49	ND	37	ND
12	ND	ND	ND	ND					

*ND* Furosemide not detected or below the lower limit of quantification (25 ng/mL) of the HPLC_UV method. No furosemide was detected at later time points

In both treatment sequence groups of 2 horses, the concentration time profiles of furosemide during the first 4 h after its administration were relatively similar, as reflected by the overlapping of individual drug concentration vs. time profiles between 0 and 3 h after drug administration. In all horses, the maximum concentration was observed in the first sample collected (0.1 h) after drug administration. The average maximum observed concentrations, AUC_0–1.5h,_ and AUC_0–3h_, of both groups of horses were not different (*p* > 0.05). For horse D in the 4 h pre-exercise treatment group, we do not report PK parameter estimates using compartmental analysis because there was not enough data for appropriate fitting of pharmacokinetic models. Thus, for this horse, we only report pharmacokinetic parameters estimated by non-compartmental analysis.

The plasma concentration of furosemide in all the horses fell below the limit of quantification (25 ng/mL) within 12 h after drug administration. In the group treated 24 h before exercise, none of the horses had detectable furosemide at the time of supramaximal treadmill exercise. In the group treated 4 h before exercise, furosemide was detected 1 h before and 2 h after supramaximal treadmill exercise in 4/4 and 3/4 horses, respectively. The pharmacokinetic parameters are presented in Table 2. The mean AUC_3-last h_ of both groups of horses were not different (*p* > 0.05).

**Table 2:** Plasma pharmacokinetic parameters of furosemide in horses treated intravenously with furosemide at 1 mg/kg (*n* = 4)

Parameter	Horses treated with furosemide 4 h before supramaximal treadmill exercise						Horses treated with furosemide 24 h before supramaximal treadmill exercise with controlled access to water					
	A	F	B	D	Geo. Mean	95% CI of Geo mean	A	F	B	D	Geo. Mean	95% CI of Geo mean
α (1/h)	5.07	4.47	4.81	N/A	4.78	4.07–5.61	5.2	5.1	4.8	4.2	4.9	4.12–5.6
β (1/h)	0.14	0.20	0.18	N/A	0.17	0.1–0.27	0.18	0.07	0.08	0.11	0.10	0.05–0.20
β _HL (h)	4.90	3.57	3.82	N/A	4.06	2.68–6.16	3.8	9.3	8.4	6.0	6.5	3.40–12.4
A (ng/mL)	5180	4990	5700	N/A	5280	4470–6230	5200	5630	5380	5850	5510	5080–6000
B (ng/mL)	151	170	179	N/A	166	134–206	167	172	190	157	171	151–193
K10 (1/h)	2.55	2.60	2.70	N/A	2.61	2.43–2.81	2.8	1.7	1.6	2.8	2.05	1.38–3.09
K12 (1/h)	2.38	1.73	1.97	N/A	2.01	1.35–3.00	2.2	3.2	3.0	1.9	2.52	1.67–3.8
K21 (1/h)	0.28	0.33	0.32	N/A	0.31	0.25–0.40	0.3	0.2	0.2	0.2	0.25	0.18–0.35
V1 (mL/kg)	188	194	170	N/A	184	156–217	173	164	180	166	170	160–183
CL_Pred_ (mL/h/kg)	442	444	402	356	409	347–482	541	240	250	323	320	177–580
Tmax (h)	0.1	0.1	0.1	0.1	0.1	N/A	0.1	0.1	0.1	0.1	0.1	N/A
Cmax (ng/mL)	3270	3370	3700	5530	3880	2630–5700	3680	3670	3530	4020	3700	340–4060
C0 (ng/mL)	5150	5040	5700	8770	6000	3980–9050	5555	5830	5390	5900	5660	5300–6050
AUC_0-inf_ (h*ng/mL)	1860	4170	3950	3130	3129	1741–5624	2280	2260	2490	2810	2450	2086–2879
AUC_last_ (h*ng/mL)	1810	1900	2050	2760	2100	1500–2840	1775	2190	2530	2630	2250	1700–3000
AUC_0_1.5h_ (h*ng/mL)	1440	1540	1650	2600	1760	1150–2690	1664	1620	1610	1850	1680	1520–1800
AUC_0_3h_ (h*ng/mL)	1600	1700	1820	2760	1920	1300–2850	1775	1820	1830	2050	1870	1690–2060

*A* Distribution intercept, *α* Distribution rate constant, *B* Post-distribution phase intercept, *β* Elimination rate constant, *CL* Systemic drug clearance, *k10, k12, and k21* Microdistribution rate constants, *N/A* No applicable -- for this horse, compartmental modeling was performed but there was no appropriate PK model fitting, *AUClast* Area under the concentration vs. time curve from time 0 h to the last sampling time, *AUC*_*inf*_ Area under the concentration vs time curve from time 0 h to infinite, *AUC*_*0–1.5h and*_ *AUC*_*0–3h*_ Partial area under the concentration vs time curve from time 0 h to 1.5 and 3 h post-drug administration

### Objective 2 determination of the plasma concentration of furosemide 24 h after its administration

Using the more sensitive analytical technique (LLOQ 1 ng/mL), furosemide was quantified in the plasma of all the horses 24 h after its administration. The results are presented in Table 3. A higher concentration of furosemide was obtained in the group of horses receiving a dose of 1 mg/kg. Within the group of horses treated with 1 mg/kg furosemide, the plasma concentration at 24 h after drug administration was numerically higher (but not significantly *p* = 0.4) when the horses were subjected to the controlled water intake protocol than when they were allowed free access to water (mean ± SD, 0.8 ± 1.3 and 0.44 ± 0.72, respectively). The tolerance limits for furosemide plasma concentration are presented in Table 3.

**Table 3:** Tolerance limit and plasma concentration (ng/mL) of furosemide from Thoroughbred horses (*n* = 6) at 24 h after furosemide administration at 1 mg/Kg or 0.5 mg/Kg to horses with controlled access to water (6 mL/kg every 4 h), with the last drink being taken 8 h before exercise or free choice water until 4 h before exercise

Horse	Treatment			
	1 mg/kg		0.5 mg/kg	
	Free choice water *n* = 6	Controlled access to water *n* = 6	Free choice water *n* = 6	Controlled access to water *n* = 6
Mean	0.44	0.80	0.22	0.35
SD	0.72	1.3	0.15	0.53
Tolerance limit	5.0	9.0	1.20	3.8

## Discussion

This study reports the disposition of furosemide in racehorses exposed to supramaximal treadmill exercise either 24 or 4 h after furosemide administration of 0.5 and 1 mg/kg body weight with and without management of their pre-exercise water consumption. Furthermore, this study confirms that furosemide, at a dosage of 0.5 and 1 mg/kg body weight, is still detectable 24 h post administration, if an appropriate LC-MS/MS method characterized by a LOQ 0.1 ng/mL is used.

Following the administration of 1 mg/kg body weight, the disposition of furosemide in our racehorses agrees well with previous findings [1, 2]. In this study, horses were exposed to both treatments (controlled access to water and supramaximal treadmill exercise 24 and 4 h post-drug administration). One of the advantages of such an experimental design is that each horse served as its own control. Moreover, it enabled us to reduce the impact of inter-individual variability on our results. The first 3 h post-furosemide administration allowed us to assess the effect of water restriction on drug disposition, whereas the 3 to 24 h time points post-administration enabled us to compare the disposition of furosemide between exercised horses and horses exposed to the controlled water intake protocol.

The water management protocol used in this study did not exert any effect on the disposition of furosemide, as reflected in the lack of differences in the AUC_0–3h_ between the two groups. This finding concurs with the results reported by Knych and colleagues [12]. This finding was unexpected, particularly when considering that furosemide has a remarkable diuretic effect during the first 3 h following its administration [4]. It is likely that the controlled water protocol used in this study did not result in a significant reduction in the circulating plasma volume, nor did it alter the elimination of the drug.

In contrast, examination of the plasma concentrations of furosemide 24 h after drug administration (Table 3) might suggest that in the horses subjected to managed water intake, the concentrations of furosemide were numerically higher than those in the horses with free access to water. The large inter-individual variability compromised the ability to detect differences, therefore, a larger number of horses should be used in further studies to confirm this observation. The effects of more restrictive controlled water intake protocols on the disposition of furosemide in horses should also be evaluated.

During the period following the first 3 h after furosemide administration, in 3 out of 4 horses belonging to the group of animals exercised 4 h after the administration of the substance, the plasma concentrations of furosemide were numerically lower (Fig. 1), probably reflecting a faster decrease in the plasma concentration of the drug. The lack of a control group (i.e., horses treated with furosemide without water restriction and not exposed to supramaximal exercise) prevented us from determining whether the difference between the groups was due to the exercise or to the effect of controlled access to water. In a study conducted by Dyke and colleagues, prolonged submaximal exercise did not alter the disposition of furosemide [1]. In addition, Knych and collaborators reported that water withholding for the 4 h before exercise did not alter the disposition of furosemide in horses [12]. Although unlikely, due to its relatively short duration (< 3 mins), the possibility that the supramaximal treadmill exercise 4 h after the administration of furosemide modified the subsequent disposition of furosemide cannot be completely ruled out.

In this study the plasma concentrations of furosemide were assessed for 24 h following the administration of the drug but, with UV detection, we were only able to detect this substance in the plasma up to 12 h after its administration. Even though these sample concentrations accounted for more than 80% of the AUC_0-∞_, this was a limitation of the analytical method. Examination of the concentration vs. time profile of furosemide (Fig. 1) suggests that furosemide should still be detectable by LC-MS/MS 24 h after its administration. In fact, in a recent study, furosemide was detected in plasma up to 36 h after the administration of 250 mg/kg intravenously to racehorses [12]. This could be relevant if race day medication was banned and horses were dosed with furosemide ≥24 h before the start of a race in jurisdictions in which the presence of any furosemide in plasma at race time was prohibited. Under these conditions racing jurisdictions would be best advised to adopt an accepted threshold for plasma furosemide rather than taking a zero tolerance stance. In order to verify this observation, we employed a more sensitive analytical technique to evaluate the concentrations of furosemide 24 h after its administration in samples collected from horses exposed to four treatment protocols. As expected, using a LC-MS/MS method characterized by an LOQ of 0.1 ng/mL, we were also able to detect and quantify furosemide in the plasma of all horses after the administration of both 0.5 mg/kg and 1 mg/kg intravenously, thereby confirming previous findings [1].

There was substantial variability in the plasma concentration of furosemide in all treatment groups 24 h after its administration. It is unknown whether the plasma concentrations of this drug and the observed inter-individual variability have any biological relevance. However, it is likely that the plasma concentrations detected are pharmacologically irrelevant, at least as far as the diuretic effects of furosemide are concerned. Drawing from results published by Tobin et al., [4]. Johansson and colleagues [2] estimated that, in horses, furosemide may have a diuretic effect at plasma concentrations as low as 70 ng/mL. This concentration is > 18 times higher than the highest plasma furosemide concentration measured in our study horses 24 h after dosing. Using the mean and SD of furosemide plasma concentration generated and the tolerance limit criteria, we found with 99% certainty that 95% of the horses would have had a plasma concentration of furosemide below 9.0 and 3.8 ng/mL 24 h after the iv administration of 1.0 or 0.5 mg/kg furosemide, respectively (Table 3). Although the concentrations obtained in this study 24 h after furosemide administration are lower than the proposed minimum concentration required to exert any diuretic effect, a recent study has indicated that the urinary whole body calcium and chloride balance remain low for 72 h after a single IV injection of 0.5 mg/kg furosemide [17]. Therefore, it would be necessary to assess whether furosemide has any other renal or extra-renal pharmacological effects that may exert an impact on horses’ athletic performance.

## Conclusion

This study provides new information about furosemide disposition in Thoroughbred horses treated intravenously with 1 mg/kg furosemide 4 and 24 h before supramaximal treadmill exercise without and with managed access to water, respectively. Our findings are comparable to those recently published by Knych et al. [12], despite the differences in experimental designs (furosemide dose, different water restriction and exercise protocol). The similarity of the findings of these two independent studies clearly increases the robustness of the results of both studies.

Furosemide was detectable in the plasma of horses exposed to supramaximal treadmill exercise with and without controlled water intake 24 h after dosing. Water restriction did not exert any apparent effect on the disposition of furosemide. It remains to be determined, however, whether the attained plasma concentration of furosemide in combination with other controlled water access protocols have any direct or indirect pharmacological effect that may affect the athletic performance of the horse.

## Methods

### Animals and management

The research protocol was approved by the Washington State University Institutional Animal Care and Use Committee (ASAF #04726).

### Study population

Six horses 4–10 years of age (6.7 ± 1.9 (mean ± SD) years) with body weights 451–547 kg (491 ± 25 kg), were included in the study. Horses were purchased from their former owners at the conclusion of their racing careers by the Department of Veterinary Clinical Sciences, Washington State University for inclusion oi the study. There were 2 females and 4 males of which 3 had been gelded. A full physical examination, a complete blood count and serum biochemistry evaluations were performed on each horse before enrollment in the study. Horses had a body condition of 5 (scale 1–10), as the study commenced within 2 weeks of their arrival from the racetrack where they had been regularly undergoing strenuous exercise in the form of fast training (i.e., breezing) and racing. Their fitness was maintained with treadmill galloping exercise 3 times per week throughout the course of the study. All horses had been diagnosed as having ≥ Grade 2 EIPH using post-race tracheoendoscopy. During the study, horses were keep in individual stalls fed a free choice mixture of grass and alfalfa hay plus a fortified grain supplement twice daily.

### Objective 1 -- experimental design

Four of the horses were randomly allocated to two treatment sequences using a 2 sequence, 2 period crossover design. Horses allocated to treatment sequence 1 received furosemide (Furosemide, VetOne, Boise, ID) 24 h before exercise and were given water in a controlled manner, as described below. After the first treatment period, horses received furosemide 4 h before exercise with water withheld until after they had exercised. Horses in treatment sequence 2, received furosemide 4 h before exercise, as described below. Following the first period, horses were treated with furosemide 24 h before exercise after which access to water was controlled. In all instances, the dose of furosemide administered was 1.0 mg/kg intravenously. This dose was selected because it has been widely studied from a pharmacological perspective, thereby facilitating comparisons between horses and with the existing literature. This would not have been possible had an absolute iv dose of 500 mg been given to every horse. Food was withheld for the last 4 h before exercise. In order to avoid a carryover effect, at least 14 days elapsed (> 10 furosemide half-lives) between treatment periods.

### Water provision protocol

In order to evaluate the potential effect of controlled water intake on the disposition of furosemide, horses receiving furosemide 24 h before exercise were given water at a rate of 6 mL/kg every 4 h after the of furosemide, with the last drink occurring 8 h before exercise. Horses given furosemide 4 h before exercise had free choice access to water until they received furosemide, after which water was withheld until the exercise was completed.

### Exercise protocol

All the horses were exercised to fatigue at the speed that had a calculated oxygen demand that was 115% of its maximum oxygen consumption (V̇O_2_max) on a treadmill inclined at 5%, 4 or 24 h after the administration of furosemide. The treadmill speed was calculated from the regression equation for the linear portion of the V̇O_2_-speed relationship that was generated for each horse 4 days before commencing the study. Fatigue was defined as the point at which a horse could no longer keep pace with the treadmill despite strong verbal encouragement.

### Blood sampling times

For horses in the group treated with furosemide 24 h before exercise, blood samples were collected immediately before dosing and 0.1, 0.5, 1, 1.5, 3, 6, 9, 12 and 24 h after dosing (pre-exercise period) and 0.1, 0.5, 1, 1.5, and 3 h post-exercise**.** For the group of horses treated with furosemide 4 h prior to exercise, blood samples were collected immediately before dosing and 0.1, 0.5, 1, 1.5, 3, 6, 9, 12 and 24 h post-dosing.

Blood samples were collected into EDTA tubes and centrifuged at 1800 *x g* for 8 min. The plasma was separated and 200 μL aliquots placed in cryovials and stored at − 80 °C until samples were analyzed.

### Quantification of furosemide in plasma for evaluation of its disposition following strenuous exercise

Analysis of furosemide in plasma samples was conducted using reversed phase HPLC. The system consisted of a 2695 separations module and a 2487 ultraviolet detector (Waters, Milford, MA). Separation was attained on a Symmetry C_18_ 3.9 × 20 mm (5 μm) with a Symmetry C_18_ guard column. The mobile phase was a mixture of (A) 20 mM potassium phosphate monobasic (Sigma-Aldrich St. Louis, MO) pH 7 and (B) acetonitrile (Waters, Milford, MA). The mixture was pumped at a starting gradient of 79% A and 21% B and was adjusted to 75% A and 25% B over 14 min, and back to initial conditions over 3 min. The drug was quantified using UV detection at 220 nm, and the flow rate was 1.0 mL/min. Furosemide was extracted from plasma samples using a solid phase extraction method. Briefly, previously frozen plasma samples were thawed and vortexed, and 100 μL of each sample was transferred to a clean test tube then 100 μL of internal standard (1 μg/mL torsemide) was added. Two hundred microliters of acetonitrile was added, then tubes were vortexed for 10 s and centrifuged for 20 min (1020 x g). The supernatant solution was transferred to a pre-wet Oasis MAX 1 cc (30 mg) extraction column (Waters, Milford, MA). The columns were eluted with 2 mL of 2% formic acid in acetonitrile:methanol (60:40) and then the eluates were evaporated to dryness under a steady stream of nitrogen gas. Samples were dissolved in 250 μL of mobile phase, and a 100 μL aliquot of each was injected into the HPLC system.

Standard curves for plasma analysis were prepared by supplementing untreated horse plasma with furosemide (Sigma-Aldrich St. Louis, MO), which produced a linear concentration range of 25–7500 ng/ml.

Average recovery was 100% for furosemide and 99% for torsemide (Sigma-Aldrich St. Louis, MO). Intra-assay variability ranged from 3.0 to 8.9% for torsemide, and from 2.6 to 10.0% for furosemide, respectively. The lower limit of quantification for furosemide was 25 ng/mL.

### Estimation of pharmacokinetic parameters of furosemide

Primary and secondary pharmacokinetic parameters were determined by non-compartmental analysis using Phoenix WinNonlin® v. 7 (Certara, Princenton, NJ). Pharmacokinetic parameters included: total body (systemic) clearance (CL), area under the concentration-first moment time curve through last sample time post-dose (AUMC), elimination half-life (t_1/2_), observed maximal concentration (Cmax), time to maximal concentration (tmax), mean residence time (MRT), and area under the plasma concentration-time curve from 0 h to last sample time (AUC_0-last h_), from 0 h to 1.5 h AUC_0–1.5h_ and from 0 to 3 h after dosing (AUC_0–3h)_. The AUCs were calculated using the linear trapezoidal rule.

### Objective 2 -- experimental design

Six horses were exposed to four different combinations of furosemide administration and water intake, each of which was initiated 24 h before exercise. A randomized Latin square crossover design was used and in no instance was there less than 14 days between any treatment protocol. *The treatments were:* 1) furosemide at 1 mg/kg intravenously pre-exercise with free choice access to water until 4 h before exercise; 2) furosemide at 1 mg/kg intravenously pre-exercise with controlled access to water (6 mL/kg every 4 h), with the last drink being taken 8 h before exercise; 3) furosemide at 0.5 mg/kg intravenously pre-exercise with free choice water until 4 h before exercise; and 4) furosemide at 0.5 mg/kg intravenously pre-exercise with the same controlled access water as described above.

### Exercise protocol

All horses were exercised to fatigue on a treadmill at the speed that had a calculated V̇O_2_ that was 115% of its V̇O_2_max as described for Objective 1.

### Blood sampling and processing

Jugular blood samples were collected into EDTA tubes 24 h after drug administration; i.e., immediately before exercise. Samples were promptly centrifuged at 1800 *x g* for 8 min. The plasma was separated and 200 μL aliquots were placed into cryovials and stored at − 80 °C until analyzed.

### Detection of furosemide concentration in plasma 24 h after the administration of furosemide using four different treatment protocols

In order to determine the concentration of furosemide in equine plasma 24 h after administering the drug, samples were submitted to LGC Science (LGC Science, Inc., Lexington, KY), because their analytical methods were characterized by a lower limit of quantification than the one used to evaluate the pharmacokinetics of furosemide (see Objective 1).

Analysis of furosemide in plasma samples was conducted using a validated liquid chromatography-mass spectrometric method (LC-MS/MS) under electrospray ionization conditions using a triple quadrupole mass spectrometer in negative ionization mode [18]. The system consisted of an Accela 1250™ (Thermo Fisher, Waltham, MA) separations module and TSQ Vantage™ mass detector (Thermo Fisher, Waltham, MA). Separation was attained on a 75 mm × 2.1 mm, 2.7 μm Ascentis Express™ (Supelco Sigma-Aldrich St. Louis, MO) and 5 mm × 2.1 mm, 2.7 μm Ascentis Express™ guard column (Supelco, (Sigma-Aldrich St. Louis, MO).

The selected reaction monitoring (SRM) transition used to quantify furosemide was m/z 331 > m/z 207 whereas, the SRM transitions used to identify furosemide were m/z 331 > m/z 287 and m/z 331 > m/z 128. Similar transitions were used to monitor the internal standard (furosemide-d_5_).

Furosemide and the internal standard were extracted using reversed-phase solid phase extraction of a proprietary water-wettable polymer (Cerex® WWP, 96, 1-mL columns in RCH, 20 mg cartridges, SPEware Corporation, Seestrasse Männedorf) by the following procedure. The cartridges were sequentially conditioned with 0.5 mL of methanol and 0.5 mL of water. Plasma samples were then loaded onto the cartridges and allowed to flow through at a rate of 1–2 mL/min The cartridges were washed with 0.5 mL of water:methanol (80:20, v/v) and the pressure was increased briefly to expel any remaining solvent in the solvent bed. The analytes were then eluted from the columns with two 500 μL aliquots of methanol. The pressure was increased briefly after each elution to expel any remaining solvent in the solvent bed. The eluates evaporated under nitrogen gas. The residues were then dissolved in 100 μL of water:methanol (80:20, v/v), vortex-mixed to assure dissolution, and the resulting solutions were transferred to glass autosampler vials for LC-MS/MS analysis.

Calibrators were prepared from negative control plasma and working standard solutions of furosemide in methanol. The calibrators were prepared in 0.5 mL of plasma each at concentrations of 0, 100, 200, 500, 1000, 2000, 5000, 7500, and 10000 pg/mL of plasma.

The method was characterized by a lower limit of quantification (LLOQ) of 0.1 ng/mL and acceptable accuracy and precision throughout the calibration range of 0.1 ng/mL to 10 ng/mL. Bias was insignificant, and the relative standard deviation of the inter-batch precision of the method at 1 ng/mL was 5.1%.

### Pharmacokinetics analysis

Compartmental and non-compartmental analyses were performed as implemented by Phoenix WinNonlin® v. 7 (Certara, Princeton, NJ). For compartmental analysis, we implemented a standard 2-stage approach (Gabrielsson and Weiner 2016). Compartmental analysis was performed with a weighting factor of 1/(observed Y), where Y was the plasma concentration. The experimental data were modelled using a 2 compartmental model. The compartmental structure was defined by standard diagnostic tools including: standard errors of the estimates, correlation matrix, and residual plots, F test, Akaike’s information criterion and Schwarz criteria.

For the 2-compartment biexponential analysis used for the data, the corresponding equation was: C = Ae^–αt^ + Be^–βt^, where C is the plasma drug concentration at time t, A and B are the y-axis intercepts for the initial and terminal phases of the curve, respectively, and α and β are the slopes of the initial and terminal phases of the curve, respectively.

Several primary and secondary pharmacokinetic parameters including total body (systemic) clearance, area under the plasma concentration-time curve from 0 h to last sample time and from 0 h to infinity after dosing, area under the concentration-first moment time curve through last sample time post-dose, elimination half-life, maximal concentration, time of maximal concentration, and rate constant of elimination were estimated. The AUC_0–1.5_, AUC_0–3_ and AUC_0-last_ were calculated by the linear trapezoidal rule. Other compartmental pharmacokinetic parameters were calculated according to formulae described elsewhere [19]. The pharmacokinetic parameters were reported as geometric mean and its 95% confidence interval.

### Statistical analysis

Descriptive statistics were used to assess the data. Normality of the distribution of the pharmacokinetic parameters was assessed using the Shapiro-Wilk test (significance level *p* ≤ 0.05). The geometric means of clearance, AUC_0–1.5h,_ AUC_0–3h_ and AUC_0-last h_ (objective 1) of each group were compared statistically using the Student t-test. The level of significance for the statistical comparisons was set at *p* ≤ 0.05. Parameters are reported using 3 significant digits.

Using the mean and standard deviation of the plasma concentration of furosemide at 24 h post-drug administration of each group, we estimated the upper tolerance intervals for 95% of the population with 99% certainty using standard methods (http://statpages.org/tolintvl.html) (NIST/Sematech Handbook Section 7.2.6.3).

## Data Availability

The datasets used and/or analysed during the current study are available from the corresponding author on reasonable request.

## Abbreviations

EIPH: Exercise-induced pulmonary hemorrhage
HPLC-UV: High performance liquid chromatography with ultra-violet detection
LC-MS/MS: Liquid chromatography with ultra-violet detection with tandem mass spectrometry detection
LLOQ: Lower limit of quantification
